# Testicular Cancer—Surgical Treatment

**DOI:** 10.3389/fendo.2019.00308

**Published:** 2019-05-15

**Authors:** Rodrigo Markus Vaz, Gustavo Bordenali, Mauro Bibancos

**Affiliations:** ^1^Department of Urology, Pontifical Catholic University of Campinas, Campinas, Brazil; ^2^Fivmed Laboratory, Department of Andrology, Fivmed Reproductive Medicine, Campinas, Brazil; ^3^Fertility Medical Group, São Paulo, Brazil

**Keywords:** testicular, cancer, surgical, treatment, lymph node, dissection, techniques

## Abstract

Testicular Germ Cell Tumor (GCT) is the most common solid tumor in men between the ages of 20–44. Men diagnosed with GCT have excellent survival rates due to advances in the multimodal treatment paradigm of chemotherapy, radiation therapy, and surgery. When considering the adequate treatment, several variables should be investigated and known to select the proper procedure. Therefore, when considering Testicular Intra-Epithelial Neoplasia, organ-sparring treatment, such as radiotherapy or organ-sparring surgery should be considered, reaching a cure rate of 98%. However, when the case is of a seminoma or a non-seminoma, orchiectomy is usually the chosen procedure, reaching an oncological cure rate of 80–85%, when there is no metastasis. Retroperitoneal Lymph Node Dissection (RPLND) is generally considered as a treatment option for non-seminomas, when lymph nodes are compromised. There are three different RPLND techniques: open, laparoscopic, and robotic. The open approach is as effective as the other two in its oncological efficiency. Although, when considering both laparoscopic and robotic approach, hospital stays are significantly reduced, better cosmetic results, and less complications when compared to the open approach. Both laparoscopic and robotic approaches require extensive experience and have a steep learning curve, while also providing similar outcome, however, recent studies have been pointing out a slight increase of advantages on the robotic approach. Therefore, further studies are necessary to assert the robotic approach superiority. Also, it is noteworthy that new technologies are on the rise, improving the laparoscopic approach, requiring further studies after their uses are consolidated.

Testicular germ cell tumor (GCT) is the most common solid tumor in men between the ages of 20 and 44. Men diagnosed with GCT have excellent survival rates due to advances in the multimodal treatment paradigm of chemotherapy, radiation therapy, and surgery (1).

Testicular cancer is divided into two large groups for treatment planning: seminoma and non-seminoma. Non-seminomatous testicular tumors include: embryonal carcinomas, yolk sac tumors, choriocarcinomas, teratomas, and mixed germ cell tumors. Teratomatous elements can be found within non-seminomas, increasing the odds of chemotherapy resistance; therefore, requiring surgical treatment for cure. However, pure seminomas do not contain such elements. Accordingly, surgical treatment plays a larger role in the treatment of non-seminomas than in the treatment of seminomas (2).

In patients suspected to have malignant cancer, radical orchiectomy is the chosen diagnostic and therapeutic procedure. History and physical, alpha-fetoprotein, beta-hCG, LDH, chemistry profile, and testicular ultrasound should be performed before the surgery. The access is made through an inguinal incision, allowing the complete removal of the ipsilateral testicle, epididymis, and spermatic cord at the height of the internal inguinal ring. The results after a single radical orchiectomy are between 80 and 85% of oncological cure, when there is no metastasis (2).

When comparing post-orchiectomy oncological outcomes, it is worth noting that non-seminomas present a higher relapse risk when compared to seminomas, especially when lymphovascular invasion has occurred (3). Therefore, post-orchiectomy surveillance is a feasible option in both cases; however, it has a greater importance when it comes to non-seminomas (3).

The International Germ Cell Cancer Consensus Group (IGCCCG) classifies patients into three different groups, depending on the place of disease and level of marker elevation: good, intermediate, or poor prognosis (4). This classification has been incorporated into the Tumor, Node, and Metastases (TNM) system. Approximately 65% of patients with metastatic non-seminomas in modern series are ranked into the good prognosis group, which has survival rates of roughly 97% when retroperitoneal lymph node dissection is performed with several different techniques, discussed below, and chemotherapy (4). The majority of patients (>95%) with metastatic seminoma are classified into the good prognosis group, having survival rates of 95% or more (4). The intermediate prognosis group include 20% of the metastatic non-seminoma and only 3% of seminomas, having an overall survival rate of about 90% (4). The only participant of the poor prognosis group, which comprises ~20% of patients with metastatic disease, is the non-seminoma, with survival rates of 65–70% (4).

Testicular Intra-epithelial Neoplasia (TIN) is considered the precursor of GCTs (5). TINs have four important and particular characteristics that directly affect its management. The first is that TIN is frequently distributed over wide areas of the affected testicle; therefore, testicular biopsies are able to provide the diagnosis. The second is that TIN is frequently present in the testicle a reasonable amount of time before the cancer progression. Third, immunohistological methods are able to safely detect TIN. And lastly, if TIN is clinically found, organ-sparring treatment is possible (5).

Considering the latter, for TIN treatment options, local radiotherapy is the safest one, with a 98% success rate (5). Another available option is chemotherapy, although it has remarkably lower efficacy, with a success rate of only 76% after three cycles (5). Another possibility is performing an organ-sparing surgery, allowing testicle preservation.

Testis-sparing surgery is predominantly considered in patients with benign lesions and TIN, with tumor mass size of 1–1.5 cm or less, and who have either only one or both testicles afflicted with the disease. In these cases, orchiectomy could be considered an overtreatment, assuming that the patient would become infertile after the procedure (6). Recent studies have shown that in these cases of small scrotal masses, testis-sparing surgery is a reliable and secure option, although some articles point out the concern of multifocal tumors; therefore, an excision contemplating a 1 cm safety rim of normal testicle tissue should be performed in addition to the tumoral mass, diminishing the risk of leaving malignant satellite lesions (7).

Retroperitoneal lymph node dissection (RPLND) has been utilized for treatment of GCTs since the 1900s, and a great amount of data is available, demonstrating its long-term efficacy and safety (4). As opposed to chemotherapy, surgery is not associated with cardiopulmonary disease, metabolic syndrome of secondary malignancy. The surgery alone reduces the probability of requiring subsequent chemotherapy by 50% and excludes the need for abdominal computed tomography (CT) scans during follow-up (4). Nonetheless, primary RPLND does not exclude the risk of recurrence outside the retroperitoneum (5–8% of all recurrences in stage I and 30% of patients with pathological stage II disease), being the lungs the most affected organ (4, 8).

It is important to note that RPLND is mostly recommended as a non-seminoma treatment option, assuming that surveillance and chemotherapy are currently the most suited options for seminomas (9). However, if the patient is not willing to undergo surveillance, chemotherapy is more effective than RPLND, in the case of non-seminomas (9).

Still considering non-seminomas, in the case of salvage treatment on patients with recurrence during surveillance, 3–4 cycles of BEP chemotherapy should be performed (9). Afterwards, the need of postchemotherapy RPLND should be evaluated individually and performed if necessary (9).

RPLND requires extensive experience, as discussed below; therefore, when performed outside centers with a high volume of surgeries, it is associated with higher morbidity and higher infield recurrence rate. Additionally, if positive lymph nodes are detected on primary RPLND, patients still need to undergo adjuvant chemotherapy of two cycles of bleomycin/etoposide/cisplatin (BEP) (4). Therefore, European and Canadian consensus guidelines no longer recommend primary RPLND for stage I Non-Seminoma Germ Cell Tumors (NSGCTs), while the National Comprehensive Cancer Network (NCCN) guidelines still list it as a valid option (2, 4, 9).

RPLND has greatly improved throughout the years, especially with the introduction of the laparoscopic approach in 1992 and recently, in 2006, with the robotic-assisted approach (10).

Laparoscopic Retroperitoneal Lymph Node Dissection (L-RPLND), on its earliest reports, provided a reduced recovery time, less blood loss and lower complications rates when compared to Open Retroperitoneal Lymph Node Dissection (O-RPLND). However, the operation had a lower lymph node yield, a very steep learning curve, and little long-term oncologic outcomes studies (10).

In 2005, Nassar Albqami and Günter Janetschek published a study comparing O-RPLND and L-RPLND in the management of Clinical Stages (CS) I and II testicular cancer, focusing on mean operation time, mean blood loss, length of stay in hospitals and relapses during follow-up as well as surgical and oncologic efficacy, complication rates, morbidity, cosmetic results, diagnostic accuracy, and recurrence rates (10).

The obtained results strongly suggested that L-RPLND, when compared to O-RPLND, provided equivalent surgical and oncologic efficiency, with similar survival and tumor-recurrence rates. However, the patient satisfaction was clearly higher with L-RPLND, because it delivers better cosmetic results, quicker convalescence, less postoperative mortality, less complications, and shorter operation times. It is noteworthy that the procedure is indeed difficult, but once the steep learning curve has been overcome, the advantages make L-RPLND better than O-RPLND (10).

Within the last decade, robotic-assisted technology has emerged in the field of urology as an alternative to the traditional laparoscopic surgery. The robot grants a greater extent of freedom of movement and better three-dimensional visualization, while still providing the perks of a minimally invasive approach. The greatest debate about the use of robotics lies in the increased cost of the technology (11).

In 2015, a study from Harris, Gorin, Ball, Pierorazio, and Allaf, from the John Hopkins' Urology Institute, published the first retrospective article comparing the results of both laparoscopic and robot-assisted approaches performed in their center from 2006 to 2014. In this period of time, 16 Robotic-Assisted Retroperitoneal Lymph Node Dissection (R-RPLND) and 21 L-RPLND were performed by a single surgeon, being all of them stage I NSGCTs (12).

The results denote that R-RPLND is equivalent to L-RPLND when comparing perioperative results and safety. Specifically, the analyzed parameters, from which all had similar values, were: complication rates, operative times, estimated blood loss, and conversions. Additionally, these parameters were also similar among groups: ejaculatory status, LN yield and frequency of LN positivity (12).

Supporters of the robotic technology state that, when analyzing prostatectomies and nephrectomies, the superior perioperative results are not the only advantage, but is also worth mentioning the improvement of intracorporeal suturing and better control around nerve plexuses and vessels. These technical improvements that the robotic technology provides are of great importance to the development of RPLND, particularly when considering the number of LNs resected and the success of nerve-sparing technique as evidenced by the protection of great vessels, nerve plexuses, and antegrade ejaculation (12).

In 2016, an article analyzing 20 R-RPLND performed on NSGCTs with clinical stages (CS) I and II and postchemotherapy, presented the advantages of the robotic-assisted approach as it is easier to be reproduced, whereas the conventional laparoscopic approach requires an experient surgeon and demands a steep learning curve. In addition, it enables bilateral access in supine position, upholding the oncological principles (13).

In 2018, a systematic review from John Hopkins' analyzed 36 articles until July 2017 comparing the three different surgical approaches (open, laparoscopic, and robotic-assisted) and concluded that the robotic-assisted approach enables equivalent or even superior oncological results compared to the other approaches when performed by experient surgeons. Moreover, the R-RPLND offers greater dexterity, superior visualization, a shorter learning curve for the surgeon and less complications overall, while still providing better recovery advantages compared to the L-RPLND, such as shorter length of stays in hospitals and reduced complication rates. However, larger prospective studies are still required to better evaluate long-term oncologic outcomes and complication rates in both the primary and post-chemotherapy settings (1).

In conclusion, further studies should be performed with a larger number of case reports to assert the superiority of the R-RPLND. It is noteworthy that the ever-growing technology evolution has been providing innovations for the conventional L-RPLND; for example, three-dimensional visualization and multi-articular clamps, which could, eventually, close the gap between L-RPLND and R-RPLND, because R-RPLND still has its disadvantages, such as lack of tactile feedback to the surgeon, inability to move the surgical table once the arms of the robot are fixed, and expenses related to the robot and its semi-disposable instruments. Therefore, further studies are required in the future to determine the best available technique, as they are currently improving (14).

## Data Availability

All datasets generated for this study are included in the manuscript and/or the supplementary files.

## Author Contributions

MB organized, supervised, corrected, and submitted the manuscript. RV and GB researched and read the bibliography used, and wrote approximately half of the manuscript.

### Conflict of Interest Statement

The authors declare that the research was conducted in the absence of any commercial or financial relationships that could be construed as a potential conflict of interest.
